# Disentangling heterogeneity in substance use disorder: Insights from genome-wide polygenic scores

**DOI:** 10.1038/s41398-024-02923-x

**Published:** 2024-05-29

**Authors:** Laura Vilar-Ribó, Judit Cabana-Domínguez, Silvia Alemany, Natalia Llonga, Lorena Arribas, Lara Grau-López, Constanza Daigre, Bru Cormand, Noèlia Fernàndez-Castillo, Josep Antoni Ramos-Quiroga, María Soler Artigas, Marta Ribasés

**Affiliations:** 1 Psychiatric Genetics Unit, Group of Psychiatry, Mental Health and Addiction, Vall d’Hebron Research Institute (VHIR), Universitat Autònoma de Barcelona, Barcelona, Spain; 2 Department of Mental Health, Hospital Universitari Vall d’Hebron, Barcelona, Spain; 3 Biomedical Network Research Centre on Mental Health (CIBERSAM), Instituto de Salud Carlos III, Madrid, Spain; 4 Departament de Genètica, Microbiologia i Estadística, Facultat de Biologia, Universitat de Barcelona, Barcelona, Catalonia Spain; 5 Department of Psychiatry and Forensic Medicine, Universitat Autònoma de Barcelona, Barcelona, Spain; 6 Addiction and Dual Diagnosis Unit, Department of Psychiatry, Hospital Universitari Vall d’Hebron, Barcelona, Spain; 7 Centro de Investigación Biomédica en Red de Enfermedades Raras (CIBERER), Instituto de Salud Carlos III, Madrid, Spain; 8 Institut de Recerca Sant Joan de Déu (IRSJD), Esplugues de Llobregat, Catalonia Spain; 9 Institut de Biomedicina de la Universitat de Barcelona (IBUB), Barcelona, Catalonia Spain

**Keywords:** Genomics, Clinical genetics, Addiction

## Abstract

Substance use disorder (SUD) is a global health problem with a significant impact on individuals and society. The presentation of SUD is diverse, involving various substances, ages at onset, comorbid conditions, and disease trajectories. Current treatments for SUD struggle to address this heterogeneity, resulting in high relapse rates. SUD often co-occurs with other psychiatric and mental health-related conditions that contribute to the heterogeneity of the disorder and predispose to adverse disease trajectories. Family and genetic studies highlight the role of genetic and environmental factors in the course of SUD, and point to a shared genetic liability between SUDs and comorbid psychopathology. In this study, we aimed to disentangle SUD heterogeneity using a deeply phenotyped SUD cohort and polygenic scores (PGSs) for psychiatric disorders and related traits. We explored associations between PGSs and various SUD-related phenotypes, as well as PGS-environment interactions using information on lifetime emotional, physical, and/or sexual abuse. Our results identify clusters of individuals who exhibit differences in their phenotypic profile and reveal different patterns of associations between SUD-related phenotypes and the genetic liability for mental health-related traits, which may help explain part of the heterogeneity observed in SUD. In our SUD sample, we found associations linking the genetic liability for attention-deficit hyperactivity disorder (ADHD) with lower educational attainment, the genetic liability for post-traumatic stress disorder (PTSD) with higher rates of unemployment, the genetic liability for educational attainment with lower rates of criminal records and unemployment, and the genetic liability for well-being with lower rates of outpatient treatments and fewer problems related to family and social relationships. We also found evidence of PGS-environment interactions showing that genetic liability for suicide attempts worsened the psychiatric status in SUD individuals with a history of emotional physical and/or sexual abuse. Collectively, these data contribute to a better understanding of the role of genetic liability for mental health-related conditions and adverse life experiences in SUD heterogeneity.

## Introduction

Substance use disorder (SUD) is a growing global health problem impacting the individual’s life and the society as a whole. In 2019, 3.2 million people died due to SUD-related causes with 300,000 deaths due to drug or alcohol overdose [1].

The presentation of SUD is highly heterogeneous across a wide range of phenotypic outcomes such as type of substance(s) [2], age at onset of SUD [3, 4], individual personality profiles [5, 6], presence of comorbid conditions [7] and disease trajectory [8]. For instance, polysubstance use, present in approximately 50% of individuals with SUD [9], has been associated with poorer treatment outcomes [10], higher rates of premature death due to overdose [2], and higher rates of mental-health problems and risky behaviours [9]. Early-onset substance users are at higher risk for psychosocial problems [3], unemployment [11], low educational attainment [4], and heavier drug abuse in adulthood [8]. The presence of comorbid psychiatric disorders has been associated with adverse disease trajectories, such as poorer treatment adherence in individuals with comorbid major depression disorder or attention-deficit hyperactivity disorder (ADHD) [10, 12], increased rates of suicide in individuals with comorbid schizophrenia [13], and worse physical and mental health in individuals with comorbid post-traumatic tsress disorder (PTSD) [14]. In addition, behavioural traits, such as neuroticism, have been associated with lower rates of abstinence and increased symptom severity [6]. Most available inpatient and outpatient treatments for SUD, however, are not well suited to accommodate the observed heterogeneity, resulting in high rates of early treatment termination and relapse [15].

Twin and adoption studies support the role of moderate to high (30–70%) genetic influence on SUD [16] and genome-wide associations studies (GWASs) have identified risk loci associated with substance-specific SUDs [17–20]. These studies, together with other genetic approaches, point to a shared genetic liability and a unitary genetic architecture of SUD across different substances [21, 22]. In addition, SUD genetic liability, which can be assessed using polygenic scores (PGSs), presents substantial overlap with psychiatric disorders and behavioural traits [23], and shows the strongest genetic correlations with ADHD, PTSD, anxiety, schizophrenia, depression, bipolar disorder and risk-taking behaviours [22, 24, 25]. Supporting this idea, a recent study in a deeply phenotyped SUD sample reported that PGSs for substance-specific SUDs were associated with their primary substance-related phenotypes but also with major depression disorder, PTSD, lifetime trauma assessment, being suspended from school or family history of SUD [26]. This finding suggests that the genetic liability for co-occurring psychopathology may explain part of the heterogeneity found in SUD.

In addition, there is growing evidence that the effect of genetic risk on SUD can be moderated by environmental factors, which may also contribute to the individual differences in addictive behaviours [27]. For instance, adverse life experiences, such as trauma exposure or peer drug use, seem to moderate the effect of PGS for cannabis use on lifetime cannabis use [28], the effect of PGS for bipolar disorder on alcohol misuse [29] and the effect of PGS for alcohol problems in adults on earlier alcohol problems [30].

In the present study, we aim to disentangle SUD heterogeneity in a SUD cohort of 1427 individuals who underwent deep phenotyping by conducting a systematic investigation of associations between 39 SUD-related phenotypes and the genetic liability for psychiatric disorders and related traits using PGSs, and to assess whether the profile of PGS associations across SUD-related phenotypes is modulated by exposure to lifetime emotional, physical and/or sexual abuse.

## Materials and methods

### Sample description

A total of 1427 individuals with SUD were recruited at the Addiction and Dual Diagnosis Unit of Hospital Universitari Vall d’Hebron, Barcelona, Spain. Inclusion criteria were age over 18 years old, substance abuse or dependence according to the DSM-IV criteria, European ancestry, and a signed informed consent prior to participation. The study was approved by the Clinical Research Ethics Committee (CREC) of Hospital Universitari Vall d’Hebron, methods were performed in accordance with the relevant guidelines and regulations and written informed consent was obtained from all subjects before inclusion in the study.

### Clinical assessment

**Fig. 1:**
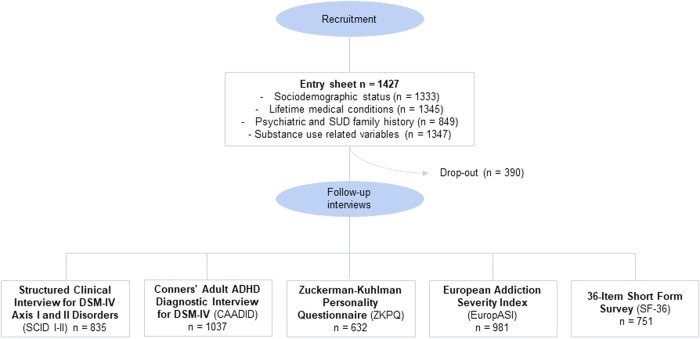
Flowchart*.* Flowchart with the different stages of the study including the recruitment and follow-up of the SUD sample. Sample size (*n*) refers to the number of individuals with at least one item of the interview available.

The clinical assessment was conducted by trained psychiatrists and psychologists in two different steps: (i) At recruitment, a questionnaire designed ad hoc was administered to gather information on sociodemographic status (sex, age, educational attainment, employment status, and criminal record), lifetime medical conditions, psychiatric and SUD family history and substance use related variables (substance(s) of use and/or abuse, age at onset of use, age at onset of SUD, years of substance use and SUD treatment history); (ii) The follow-up interviews were divided into four sessions to evaluate SUD severity, DSM-IV axis I and axis II disorders, heath-related quality of life and personality traits with different scales and questionnaires (Fig. 1), detailed below.

The Structured Clinical Interview for Axis I and II Disorders of the DSM-IV (SCID-I and SCID-II) [31] and the Conners’ Adult ADHD diagnostic interview for DSM-IV (CAADDID-II) [32] were used to assess psychiatric comorbidity. The Spanish version of the Zuckerman–Kuhlman Personality Questionnaire (ZKPQ) [33] was used to assess personality features including neuroticism-anxiety, activity, sociability, impulsive sensation-seeking, and aggression-hostility. The validated Spanish version of the European Addiction Severity Index interview (EuropASI) [34] is designed to provide information about aspects of an individual’s life that may contribute to his/her substance abuse, specifically in the following areas: legal status, employment status, medical status, psychiatric status, drug use, alcohol use, and family/social relationships. Scores ranging from 0 to 1 are estimated, with higher scores indicating greater severity. The 36-item Short Form Survey (SF-36) was administered to measure self-reported health and quality of life, both physically and mentally with higher scores indicating better health [35]. After data curation, 39 phenotypes with a sample size >300 were considered and classified into three categories: SUD variables (*n* = 8), comorbidity and personality traits (*n* = 15), and sociodemographic and health outcomes (*n* = 16) (Table 1). For binary traits, with n1 individuals in category one and n2 individuals in category two, the effective sample size was calculated with the formula 4/(1/n1 + 1/n2).

**Table 1:** Summary of the 39 SUD-related phenotypes.

Phenotypes	*n*^a^	Summary
Sex: Male (%)	1092	76.5
Age; Mean (SD)	1427	38.6 (10.3)
**SUD variables**		
Age at onset of substance use; Mean (SD)	1347	17.05 (1.36)
Age at onset of SUD; Mean (SD)	1339	19.32 (1.4)
Years between substance use and SUD; Median (IQR)	1325	1 (3)
Years of substance use as proportion of lifespan; Median (IQR)	1148	34 (33)
Number of substances consumed	869	
1 (%)		23.2
2 (%)		29.5
3 or more (%)		47.3
Number of therapeutic community interventions; Median (IQR)	1314	
0 (%)		64.5
1 (%)		22.7
2 (%)		7.1
3 or more (%)		5.7
Number of inpatient detoxifications; Median (IQR)	1327	
0 (%)		70.5
1 (%)		16.0
2 (%)		6.8
3 or more (%)		6.7
Number of outpatient treatments; Median (IQR)	1252	
0 (%)		28.5
1 (%)		37.9
2 (%)		16.6
3 or more (%)		17
**Comorbidity and personality traits**		
*Mental disorders in DSM-IV*		
Borderline personality disorder (%)	447	15.9
Major depressive disorder (%)	828	37.8
Antisocial personality disorder (%)	560	21.3
Psychotic disorder (%)	593	7.8
Anxiety disorder (%)	654	24.9
Attention deficit hyperactivity disorder (%)	700	21.5
*Zuckerman–Kuhlman Personality Questionnaire (ZKPQ)*		
Neuroticism Anxiety personality factor; Mean (SD)	663	10.8 (4.9)
Aggression Hostility personality factor; Mean (SD)	667	8.93 (3.15)
Sociability personality factor; Mean (SD)	632	6.6 (3.4)
Impulsive sensation seeking personality factor; Mean (SD)	666	10.5 (4.3)
Activity personality factor; Mean (SD)	665	8.09 (3.5)
Suicide attempt (%)	618	47.8
Suicide ideation (%)	731	30.1
Psychotic symptoms (%)	1281	58.6
Sleeping disturbances (%)	1228	54.6
**Sociodemographic and health outcomes**		
* EuropASI*		
Legal status; Median (IQR)	984	0 (0.1)
Employment status; Median (IQR)	984	0.55 (0.5)
Medical status; Median (IQR)	982	0 (0.1)
Psychiatric status; Median (IQR)	984	0.4 (0.3)
Drug use; Median (IQR)	984	0.2 (0.2)
Alcohol use; Median (IQR)	984	0.1 (0.3)
Family/Social relationships; Median (IQR)	981	0.4 (0.5)
*36-Item Short Form Survey (SF-36)*		
Physical health; Mean (SD)	751	48.4 (1.8)
Mental health; Mean (SD)	751	35.5 (13.7)
Criminal record (%)	713	43.1
Unemployment (%)	1057	73.4
Number of psychiatric hospitalizations; Median (IQR)	760	
0 (%)		79.9
1 (%)		9.9
2 (%)		4.5
3 or more (%)		5.7
Psychiatric family history (%)	840	44.8
Lifetime medical conditions (%)	1334	54.4
Substance use family history (%)	818	59.6
Educational attainment	1333	
1 (Incomplete primary school) (%)		14.5
2 (Primary school) (%)		40.9
3 (Secondary/High school) (%)		35.6
4 (Bachelor’s degree or higher) (%)		9.1

^a^For binary traits, with n1 individuals in category one and n2 individuals in category two, effective sample size was calculated with the formula 4/(1/n1 + 1/n2).

### Genotyping and quality control

Genomic DNA was isolated from whole blood by the salting-out procedure and genotyped with the Illumina Infinium Global Screening Array-24 version 2 (GSA v2) (Illumina, CA, San Diego, USA) in two different waves (434 and 993 samples, respectively). Pre-imputation quality control was done with the PLINK 2.0 software [36] and included individual and variant filtering based on the following parameters: variant call rate > 0.95 (before individual filtering), individual call rate > 0.98, autosomal heterozygosity deviation (|Fhet| < 0.2), variant call rate >0.98 (after individual filtering), SNP Hardy-Weinberg equilibrium (HWE) (*p* > 1e−10 in cases) and minor allele frequency (MAF) > 0.01. Genetic outliers were identified by principal component analysis (PCA) using PLINK 2.0 and the mixed ancestry 1000G reference panel [37]. Non-European individuals were excluded if their principal component (PC) values for PC1 and PC2 were greater than 1 standard deviation from the mean-centering point for the study population. Related and duplicated samples were identified by the “KING-robust kinship estimator” analysis in PLINK 2.0 [38] and one individual was excluded from each pair of subjects with a kinship coefficient > 0.0442. Imputation was done with McCarthy tools, for data preparation, and the Michigan Imputation Server [39], using the Haplotype Reference Consortium (HRC Version r1.1 2016) reference panel (GRCh37/hg19). Variants were excluded in case of allele mismatch between the reference panel and the study dataset (chi2 > 900). Post-imputation dosage files with imputation INFO score >0.8 and MAF > 0.01 were considered for subsequent analyses.

### Polygenic scores

Polygenic scores (PGSs) were constructed in our cohort using the PRS-CS software [40], PLINK 2.0, and available GWASs summary statistics (Table S1), on psychiatric disorders (ADHD [41], anxiety (http://www.nealelab.is/uk-biobank/), bipolar disorder [42], depression [43], PTSD [44] and schizophrenia [45]), behavioural traits (risk tolerance [46] and suicide attempt [47]) and other related traits (educational attainment [48] and well-being [49]). PGSs were computed and standardized to a mean of 0 and a standard deviation of 1 for all disorders and traits.

### Statistical analysis

#### Association between polygenic scores and SUD-related phenotypes

The profile of PGSs associations across the SUD-related traits was assessed with the appropriate regression models depending on the nature of the outcome variable with R: logistic regression for binary variables, linear regression for continuous variables, ordinal regression for ordinal categorical variables, and negative binominal for count variables. Prior to the analysis, logarithmic transformations were applied to continuous variables not following a normal distribution (“age at onset of substance use” and “age at onset of SUD”). Additionally, linear regression residuals were checked for continuous variables with significant results to ensure they followed a normal distribution. Age, sex, genotyping batch, and the 10 first PCs were included as covariates in all analyses. Additionally, for the variables “age at onset of substance use”, “age at onset of SUD”, “years between substance use and SUD”, and “years of substance use as a proportion of lifespan”, the main drug of use, abuse or dependence was included as a covariate. *P*-values were corrected for multiple comparisons using *PhenoSpD* [50, 51], a command line R-based tool for estimating phenotypic correlations and multiple testing correction. The effective number of independent variables estimated was 35 using the VeffLi model and the corrected *p*-value threshold was set at *p* < 1.46e−03 [52].

#### Interaction between polygenic scores and emotional, physical, and/or sexual abuse in SUD-related phenotypes

For those PGSs associated with any outcome, interaction with emotional, physical and/or sexual abuse was tested in a subset of 735 individuals who had completed the EuropASI family/social relationships questionnaire, and information on emotional, physical, and/or sexual abuse was available. Potential interaction effects were tested by introducing an interaction term (PGS*abuse) in the regression model adjusted for age, sex, genotyping batch, and the 10 first PCs as covariates. Multiple comparison corrected *p-*value, calculated with *PhenoSpD* in R, was set at *p* < 2.05e−03 [50, 51]. For significant interactions, PGS-outcome associations were stratified by exposure to emotional, physical, and/or sexual abuse.

#### Cluster analysis

Three cluster analyses were run considering complete cases from each category of clinical variables separately (SUD-related variables, comorbidity and personality traits, and sociodemographic and health outcomes) and hierarchical agglomerative clustering (HC) with Ward’s criterion using the *factoextra* R package [10]. To accommodate the mixed data types (including binary, quantitative, and ordinal variables) in our clinical dataset, we measured distances between observations using the Gower coefficient with the *DAISY* function implemented in the *cluster* R package [11]. We considered the number of clusters (K) ranging from 2 to 5 and those showing the maximum silhouette width (SW) were selected as optimal [12]. Cluster stability was measured with the mean of Jaccard similarities, where a mean greater than 0.6 was deemed as stable [13]. Variables were compared between clusters using different statistical tests depending on the nature of the data and the number of groups to compare: chi-square for categorical variables; T-student (two-groups) or ANOVA (more than two groups) for normally distributed continuous variables with equal variances; Mann-Whitney (two-groups) or Krushal-Wallis H (more than two groups) for non-normally distributed continuous variables; and Welch tests for normally distributed continuous variables without equal variances (Table S2). The association between PGS and clinical clusters was assessed as described above, using cluster 1 in each category as the reference group.

## Results

Our cohort consisted of 1427 individuals (76.5% male), with a mean age of 38.6 years (SD = 10.3) (Table 1). The vast majority of subjects were polysubstance users and 47% fulfilled SUD criteria for three or more substances.

### Polygenic scores for psychiatric disorders

**Fig. 2:**
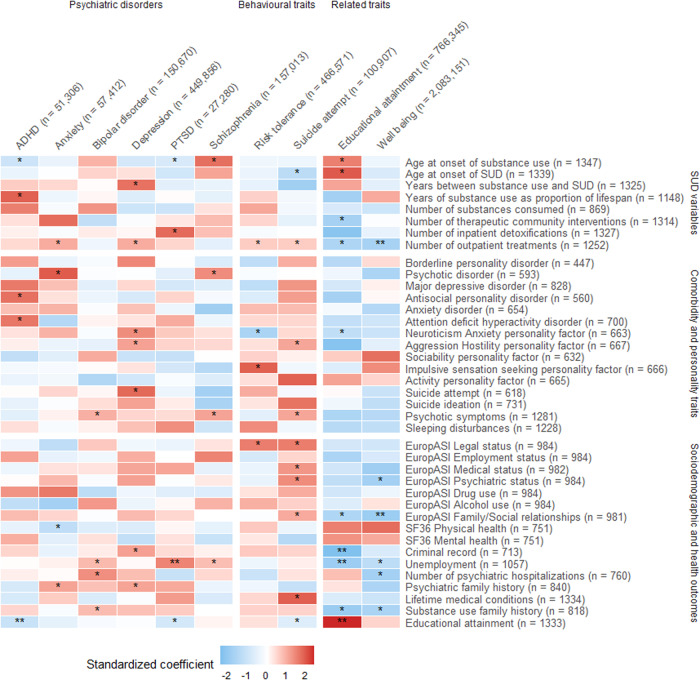
Heatmap for the results of the association between PGSs and SUD-related phenotypes. Association pattern between 10 PGSs for psychiatric disorders, behavioural and related traits with the SUD-related phenotypes; *nominal significance *p-*values; **p-values that passed multiple testing correction using PhenoSpD (*p* < 1.46−03). ADHD Attention-deficit hyperactivity disorder, PTSD Post-traumatic stress disorder. The standardized coefficient corresponds to Beta for continuous variables, log(OR) for binary and ordinal variables, and log(IRR) for count variables.

After multiple testing corrections, we found significant associations between the PGS for ADHD and lower educational attainment (odds ratio (OR) = 0.85, 95% CI [0.93, 0.77], *p* = 1.20e−03) and between the PGS for PTSD and unemployment (OR = 1.23, 95% CI [1.09, 1.40], *p* = 1.00e−03) (Fig. 2, Table S3a and S3e).

Despite not surpassing the multiple testing correction threshold (*p* < 1.46e-03), we found additional nominal associations (*p* < 0.05) . PGS for ADHD was associated with early-onset first substance use (Beta (β) = −0.01, 95% CI [−0.03, −3.00e−04]), longer-term substance use as a proportion of the lifespan (β = 1.07, 95% CI [0.03, 2.11]), lifetime diagnosis of ADHD (OR = 1.24, 95% CI [1.06, 1.45]), and antisocial personality disorder (OR = 1.24, 95% CI [1.04, 1.47]) (Fig. 2, Table S3a). PGS for anxiety was associated with more outpatient treatments (incidence rate ratio (IRR) = 1.09, 95% CI [1.03, 1.15]), psychotic disorders across the lifetime (OR = 1.49, 95% CI [1.16, 1.92]), poorer self-perceived physical health status measured with the SF-36 instrument (β = −0.93, 95% CI [−1.64, −0.22]) and psychiatric family history (OR = 1.16, 95% CI [1.01, 1.33]) (Fig. 2, Table S3b). PGS for bipolar disorder was associated with higher rates of psychotic symptoms (OR = 1.14, 95% CI [1.02, 1.28]), unemployment (OR = 1.14, 95% CI [1.01, 1.29]), psychiatric hospitalizations (IRR = 1.26, 95% CI [1.03, 1.53]) and substance use family history (OR = 1.15, 95% CI [1.0, 1.32]) (Fig. 2, Table S3c). PGS for depression showed association with slower transition from substance use to SUD (IRR = 1.1, 95% CI [1.01, 1.21]), more outpatient treatments (IRR = 1.09, 95% CI [1.03, 1.15]) and higher rates of neuroticism-anxiety (β = 0.36, 95% CI [4.00e−03, 0.72]) and aggression-hostility (β = 0.27, 95% CI [0.04, 0.5]) according to the ZKPQ, suicide attempts (OR = 1.18, 95% CI [1.02, 1.37]), criminal records (OR = 1.27, 95% CI [1.09, 1.48]) and psychiatric family history (OR = 1.16, 95% CI [1.01, 1.32]) (Fig. 2, Table S3d). PGS for PTSD was associated with early-onset of first substance use (β = −0.01, 95% CI [−0.03, −1.00e−03]), more inpatient detoxifications (IRR = 1.13, 95% CI [1.01, 1.27]), and lower educational attainment (OR = 0.87, 95% CI [0.97, 0.79]) (Fig. 2, Table S3e). And lastly, PGS for schizophrenia was associated with later onset of substance use (β = 0.01, 95% CI [3.00e−04, 0.03]), psychotic disorders (OR = 1.37, 95% CI [1.05, 1.78]), higher rates of psychotic symptoms (OR = 1.15, 95% CI [1.03, 1.29]) and unemployment (OR = 1.13, 95% CI [1.00, 1.28]) (Fig. 2, Table S3f).

### Polygenic scores for behavioural traits

None of the associations between PGSs for behavioural traits and SUD-related phenotypes surpassed multiple testing corrections, however, nominally significant associations (*p* < 0.05) are detailed bellow.

PGS for risk tolerance showed associations with more outpatient treatments (IRR = 1.06, 95% CI [1.00, 1.12]), lower rates of neuroticism-anxiety (β = −0.51, 95% CI [−0.88, −0.14]) and higher rates of impulsive sensation seeking (β = 0.32, 95% CI [3.00e−03, 0.65]) according to the ZKPQ and higher rates of legal problems measured by the EuropASI index (OR = 1.18, 95% CI [1.02, 1.36]) (Fig. 2, Table S3g). PGS for suicide attempt was associated with early-onset of SUD (β = −0.02, 95% CI [−0.04, −0.01]), more outpatient treatments (IRR = 1.06, 95% CI [1.00, 1.12]), higher rates of aggression-hostility (β = 0.26, 95% CI [0.02, 0.51]) and psychotic symptoms (OR = 1.15, 95% CI [1.03, 1.29]), more legal (OR = 1.18, 95% CI [1.01, 1.37]), medical (OR = 1.14, 95% CI [1.00, 1.29]), psychiatric (OR = 1.13, 95% CI [1.01, 1.27]) and family/social (OR = 1.13, 95% CI [1.00, 1.26]) problems measured by the EuropASI index, more lifetime medical conditions (OR = 1.14, 95% CI [1.00, 1.29]) and lower educational attainment (OR = 0.90, 95% CI [1.00, 0.81]) (Fig. 2, Table S3h).

### Polygenic scores for educational attainment and well-being

After multiple testing correction, we found significant associations between PGS for educational attainment and less criminal records (OR = 0.67, 95% CI [0.57, 0.78], *p* = 8.03e−07), unemployment (OR = 0.82, 95% CI [0.71, 0.92], *p* = 1.34e−03) and higher educational attainment (OR = 1.39, 95% CI [1.54, 1.25], *p* = 3.34e−10), as well as associations between PGS for well-being and less outpatient treatments (OR = 0.91, 95% CI [0.86, 0.96], *p* = 7.00e−04) and family/social problems (OR = 0.83, 95% CI [0.74, 0.93], *p* = 1.00e−03) (Fig. 2, Table S3i and S3j).

Nominal associations (*p* < 0.05) include the association between PGS for educational attainment and later onset of substance use (β = 0.02, 95% CI [0.01, 0.03]) and SUD (β = 0.02, 95% CI [4.00e−03, 0.04]), less therapeutic community interventions (OR = 0.89, 95% CI [0.81, 0.98]) or outpatient treatments (OR = 0.91, 95% CI [0.87, 0.97]), lower rates of neuroticism-anxiety (β = −0.40, 95% CI [−0.78, −0.01]), less social-familiar problems (OR = 0.86, 95% CI [0.77, 0.96]), and substance use family history (OR = 0.80, 95% CI [0.70, 0.92]) (Fig. 2, Table S3i). Moreover, PGS for well-being showed association with lower rates of psychiatric problems (OR = 0.87, 95% CI [0.78, 0.97]), less unemployment (OR = 0.88, 95% CI [0.77, 0.99]), psychiatric hospitalizations (OR = 0.78, 95% CI [0.68, 0.95]), and substance use family history (OR = 0.83, 95% CI [0.77, 0.99]) (Fig. 2, Table S3j).

### Interaction between polygenic scores and emotional, physical and/or sexual abuse on SUD-related phenotypes

**Fig. 3:**
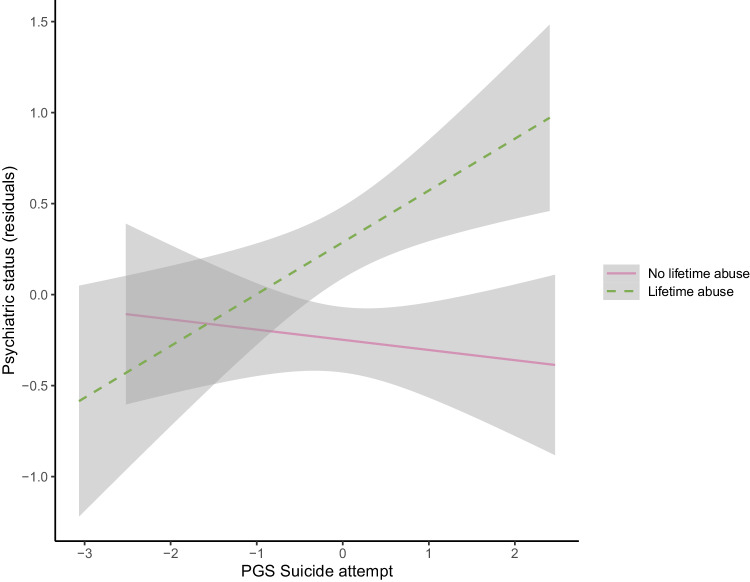
Statistically significant result from the interaction analysis*.* Interaction between PGS for suicide attempt and lifetime emotional, physical and/or sexual abuse in psychiatric status measured by the EuropASI index. The X-axis presents the PGS for a suicide attempt, and the Y-axis shows the residuals from an ordinal regression model with psychiatric status as outcome adjusted for age, sex, genotyping batch, and the 10 first principal components for those individuals who suffered lifetime abuse (in green) and those who did not (in pink).

Information on lifetime emotional, physical, and/or sexual abuse was available for a total of 735 individuals with SUD, 45.6% of which (*n* = 335) reported having experienced some sort of abuse across their lifetime. PGS*abuse interaction analysis was performed for those PGSs nominally associated with any outcome (Table S4). We found one significant interaction where lifetime abuse modified the association between PGS for suicide attempts and the psychiatric status measured by the EuropASI index (OR = 1.35, 95% CI [1.03, 1.78], *p* = 2.94e−02). Stratified results showed that the genetic liability for suicide attempt was associated with worse psychiatric status scores among those having experienced lifetime emotional, physical and/or sexual abuse (OR = 1.33 95% CI [0.48, 0.09], *p* = 4.67e−04), while the association was not significant for those not exposed (Fig. 3).

### Cluster analysis

To better understand the relationships among the phenotypic variables studied, we ran a cluster analysis and identified clusters of SUD individuals that exhibit differences in their phenotypic profile considering the three categories of outcomes separately: SUD-related variables (considering 8 variables for 665 individuals), comorbidity and personality traits (15 variables, 349 individuals) and sociodemographic and health outcomes (16 variables, 307 individuals). Cluster stability was estimated as greater than 0.60 for all clusters with the Jaccard means (Table S2).

When considering the SUD-related variables, the maximum average silhouette width (SW = 0.26) was found for two clusters (Figure S1a, Table S2). Cluster 2, which included 492 patients (74%), was characterized by a more severe course of SUD (i.e., earlier onset of first substance use, earlier onset of SUD, or faster transition from substance use to SUD, among others). For comorbidity and personality traits, two clusters were identified (SW = 0.14) (Figure S1b, Table S2), with cluster 2, which included 134 individuals (38.4%), showing higher rates of co-occurring psychiatric disorders and being nominally associated with the PGS for risk tolerance (*p* = 8.40e−03, Table S5). Finally, when considering sociodemographic and health outcomes, we estimated three clusters (SW = 0.16) (Figure S1c, Table S2). Cluster 2 and 3, which included 140 individuals (45.6%) and 107 (34.8%) respectively, showed poorer outcomes than individuals in cluster 1 in areas related to employment, psychiatric status, and self-perceived physical health (Figure S1c, Table S2). Cluster 2 also showed higher rates of medical problems, and cluster 3 showed increased rates of criminality and legal problems (Figure S1c, Table S2). Cluster 3 was also nominally associated with higher PGS for risk tolerance (*p* = 1.40e−02), depression (*p* = 3.00e−02), and suicide attempt (*p* = 4.20e−03) and lower PGS for educational attainment (*p* = 1.80e−03) (Table S5).

## Discussion

There is immense clinical and genetic heterogeneity among individuals with SUD, and current treatment approaches fail to accommodate this variability, resulting in poor treatment adherence and high rates of relapse [53]. In this study, we utilized multidimensional data from a deeply phenotyped SUD cohort and individual genetic liability information for a broad range of mental health-related traits using PGSs, to provide new insights into the heterogeneity of the disorder. Our approach included the systematic association of 10 PGSs for psychiatric disorders, behavioural and other related traits with 39 SUD-related phenotypes and clusters, and the assessment of PGS-environmental interactions using the information on emotional, physical, and/or sexual abuse. Our main findings suggest that the genetic liability for ADHD, PTSD, and suicide attempts, in conjunction with environmental factors, may underlie, at least partially, the observed heterogeneity in SUD-related phenotypes such as educational attainment, unemployment, and psychiatric status.

PGSs analysis on the SUD-related phenotypes builds on previous findings supporting links between the genetic risk for psychiatric disorders and a wide variety of SUD outcomes. In line with this evidence, our results suggest that the genetic liability for mental health-related traits exhibits different patterns of associations with SUD-related phenotypes. Specifically, we replicated previous findings linking the genetic liability for ADHD with lower educational attainment [54], the genetic liability for PTSD with higher rates of unemployment [55], and the genetic liability for higher educational attainment with lower rates of criminal records and unemployment [56, 57]. While these associations were described in the general population, our results suggest that these patterns remain in individuals with SUD. Moreover, our findings showed that the genetic liability for well-being is associated with better outcomes, namely lower rates of outpatient treatments and fewer problems related to family and social relationships, which is consistent with the role of the genetics underlying well-being in healthy family relationships [58].

Despite not surpassing multiple comparison corrections, we found evidence supporting previously reported associations. For instance, PGSs for ADHD, schizophrenia, and educational attainment were associated with their respective primary phenotype, confirming the validity of the approach. In addition, we identified an association between the genetic liability for depression and higher rates of suicide attempts. This is consistent with previous findings linking PGSs for depression with suicide attempts [59–62], and studies suggesting an increased risk of suicide attempt and ideation among individuals with comorbid SUD and major depressive disorder [63, 64]. Our findings add to the evidence supporting that genetic liability for depression may have a relevant role regarding suicide attempts and ideation in the context of SUD.

Our results also shed light on the association between the genetic liability for multiple psychiatric disorders and poor SUD-related outcomes. These include early age at the onset of substance use and a high number of outpatient treatments, strengthening the notion that genetic susceptibility to psychiatric diseases and behavioural traits may play a role in promoting the initiation and impeding the cessation of substance use [12, 65–67]. For instance, we found that individuals with higher PGS for ADHD showed earlier onset and more prolonged substance use, while those with higher PGS for depression showed faster transition from substance use to SUD and more outpatient treatments. Similarly, individuals with higher PGS for PTSD showed earlier onset of substance use and more inpatient treatments.

Moreover, PGSs for educational attainment or suicide attempts were associated with multiple outcomes (more than ten). Increased genetic risk for educational attainment was associated with fewer therapeutic interventions, late age at onset of substance use or SUD, and less SUD family history or problems related to family and social relationships. These findings are consistent with previous evidence showing that the genetic liability for education attainment is linked to decreased SUD severity [68] and a recent study by Kinreich et al. [69], suggesting that polygenic liability to years of education could be used to predict remission in patients with alcohol use disorder. Additionally, the genetic liability for suicide attempt showed the strongest association with early age at the onset of SUD, number of outpatient treatments, higher rates of psychotic symptoms, and a wide range of medical, psychological and legal problems. Adding to this evidence, we report a significant interaction between PGS for suicide attempts and having been exposed to lifetime emotional, physical, and/or sexual abuse in the psychiatric status of SUD individuals. While it is well established that exposure to sexual trauma and/or abuse increases the risk for substance use and mental health problems later in life [70], we found that the genetic liability for suicide attempts exacerbates the negative impact on mental health problems in individuals with a history of abuse. Similar findings have been reported for cannabis use [28] or bipolar disorder [71], where exposure to trauma and/or maltreatment potentiates the polygenic risk for these disorders. Overall, these results highlight that focusing on exposed individuals may render genetic effects that may not be found when environmental exposures are not considered.

Although many results are well supported by prior research, we also found that for some disorders PGSs were not associated with their primary phenotype. For instance, PGSs for anxiety or depression did not show an association with anxiety disorder or major depressive disorder in the SUD dataset. The reasons for this lack of association may include selection bias, complex relationships between SUD and comorbid conditions, and limited sample size for some of the outcomes. Moreover, PGS for suicide attempts was not associated with suicide behaviours, namely suicide ideation, and attempt, in our SUD dataset. Suicide attempt is a clinically complex phenotype that can vary greatly in frequency and intensity [72]. Even though the GWAS meta-analysis used to construct PGS for suicide attempts aimed to harmonize data across various cohorts by including clinical samples from major psychiatric disorders and individuals from the Million Veterans Project sample [47], differences in population characteristics or assessment methods of the phenotype may account for the inconclusive results observed in our dataset. In previous studies, the reliability of PGS-based predictions of the suicide attempt has been inconsistent when applied to independent datasets [73–75], and Lannoy et al. [76], found evidence for the interaction between PGS for suicide attempt and drug use on suicide ideation. Together, these results highlight the multifactorial nature of suicide attempts and suggest that other factors, such as psychiatric comorbidity, SUD type or severity, and environmental factors, should be taken into account when assessing suicide risk.

We identified clusters of individuals that exhibit differences in their phenotypic profile and in clinically relevant outcomes, including disease onset and progression, rates of comorbid disorders, physical and mental health outcomes, as well as various sociodemographic characteristics such as unemployment and criminality rates. These results highlight distinct groups of patients at high risk of poorer outcomes and suggest that the combination of key aspects of the disorder may provide clinically useful stratification for intervention and implications for prognosis and management. Further studies considering the overall range of clinical outcomes in a single cluster analysis may help to obtain a more comprehensive understanding of SUD heterogeneity. Unfortunately, this was not feasible in the present study due to the attrition in sample size for complete cases (14%; *N* = 199). Although no clear associations were found between the genetic liability for multiple mental health-related traits and the identified clusters, some of the results are consistent with previous findings and are worthy of further investigation. Specifically, we found suggestive evidence of association between the genetic liability for risk tolerance and two clusters: one predominantly composed of SUD individuals with comorbid psychiatric disorders and high rates of suicide behaviors, and the other characterized by unemployment and criminality outcomes. These findings are consistent with previous research indicating that comorbid psychiatric disorders, particularly major depressive disorder, increase the risk of suicide in individuals with SUD through risk-taking behaviors [45–47].

It is important to be cautious when comparing results from PGSs for the disorders and traits tested, taking into consideration the variations in statistical power between some of them. The differences in sample size among the GWAS meta-analyses used to construct PGSs, as well as among the outcomes, could have contributed to the uneven pattern of associations observed. Additionally, other factors such as environmental factors and sex differences may play a significant role in certain aspects of SUD heterogeneity. Furthermore, our results suggest that the patterns of lifetime comorbidity in SUD result, in part, from the contribution of genetic factors. However, it is currently unknown whether substance use is a consequence of underlying psychiatric disorders or whether it increases the risk of mental health problems later in life. Access to longitudinal data would provide new and valuable information to assess causal relationships between SUD and comorbid conditions and to examine the impact of genetic liability on disease progression.

This study supports that the genetic liability for distinct mental health-related traits plays a role in the heterogeneity of SUD and can influence disease outcomes in terms of severity, comorbidity rates, and socio-demographic factors. There is also evidence for PGS-environment interactions between the genetic liability for suicide attempts and lifetime emotional physical and/or sexual abuse on the psychiatric status of individuals with SUD. These results encourage the use of PGSs and gene-environment interactions to better understand the heterogeneity of SUD and complex traits.

### Supplementary information

Supplementary Material

Supplementary Table 3

## Data Availability

Raw data from this article is not publicly available because of limitations in ethical approvals and the summary data will be available from the corresponding author upon reasonable request.
