# Supplementary material for: Disentangling heterogeneity in substance use disorder: Insights from genome-wide polygenic scores
Source: Transl Psychiatry. 2024 May 29;14:221. doi: 10.1038/s41398-024-02923-x (PMC11137038; doi:10.1038/s41398-024-02923-x)
Supplement: Supplementary file 1 — Supplementary Material [file 41398_2024_2923_MOESM1_ESM.docx]

**Disentangling heterogeneity in Substance Use Disorder: Insights from Genome-Wide Polygenic Scores**

**Supplementary Material:**

**Figure S1.** Cluster analysis according to the SUD phenome considering three categories of clustering variables separately ..……………………………………………………………………………………………………………………… 2

**Table S1.** Discovery GWASs of psychiatric diseases, behavioral and related traits used to construct the PGSs ………………………………………………………………………………………….…………………………………………………… 4

**Table S2.** Descriptive summary table of the clusters considering the three categories of clinical variables ………………………………………………………………………………………………………………………………………… 5

**Table S4.** Interaction between PGSs and lifetime emotional. physical and/or sexual abuse in the SUD phenome ………………………………………………………………………………………………………………………………...….…… 8

**Table S5.** Results from the association between the PGSs and the clinical clusters …………………….…….. 11

**Supplementary Figure S1. Cluster analysis according to the SUD phenome considering three categories of clustering variables separately**: **(A)** SUD-related variables (665 individuals), **(B)** comorbidity and personality traits (349 individuals) and **(C)** sociodemographic and health outcomes (307 individuals). For each one, figure (i) shows the distribution of individuals across clusters, and figure (ii) shows the distribution of variables showing statistical significance per cluster. Boxplots show mean values of continuous variables, and barplots show percentages for binary variables.

**Supplementary Table 1.**

*Discovery GWASs of psychiatric diseases, behavioral and related traits used to construct the PGSs*

| **Trait** | **n cases** | **n controls** | **n total ^a^** | ***ref*** |
| --- | --- | --- | --- | --- |
| Attention-Deficit Hyperactivity Disorder | 20,183 | 35,191 | 51,306 | [1] |
| Anxiety | 16,730 | 101,021 | 57,412 | http://www.nealelab.is/uk-biobank/ |
| Bipolar Disorder | 41,917 | 371,549 | 150,670 | [2] |
| Depression | 170,756 | 329,443 | 449,856 | [3] |
| Post-Traumatic Stress Disorder | 9,354 | 25,175 | 27,280 | [4] |
| Schizophrenia | 67,390 | 94015 | 157,013 | [5] |
| Risk tolerance | - | - | 466,571 | [6] |
| Suicide attempt | 26,590 | 492,022 | 100,907 | [7] |
| Educational attainment | - | - | 766,345 | [8] |
| Well being | - | - | 2,083,151 | [9] |

^a^ For binary traits effective sample size was calculated with the formula 4/(1/n cases+1/n controls)

**Supplementary Table 2.**

*Descriptive summary table of the clusters considering the three categories of clinical variables*

| **SUD-related variables (n=665)** | **Cluster 1** | **Cluster 2** |  | **Statistical test** | ***p*** |
| --- | --- | --- | --- | --- | --- |
|  | (n=173; JM=.61) | (n=492; JM=.76) |  |  |  |
| Age at onset of substance use ^a^; mean (SD) | 25.22 (11.89) | 15.83 (3.17) | - | T-test | **5.10E-30** |
| Age at onset of SUD ^a^; mean (SD) | 32.2 (1.07) | 17.12 (3.44) | - | T-test | **1.20E-80** |
| Years between substance use and SUD; median (IQR) | 6.00 (11.00) | .00 (2.00) | - | MWU | **5.70E-22** |
| Years of substance use as proportion of lifespan; median (IQR) | 16.00 (23.00) | 39.00 (29.00) | - | MWU | **8.70E-27** |
| Number of substances consumed; median (IQR) | 2.00 (2.00) | 3.00 (2.00) | - | MWU | **4.10E-14** |
| Therapeutic community interventions ^b^ (%) | 17.3 | 46.1 | - | χ^2^ | **4.11E-11** |
| Inpatient detoxifications ^b^ (%) | 17.9 | 31.7 | - | χ^2^ | **7.48E-04** |
| Outpatient treatments ^b^ (%) | 64.2 | 73.6 | - | χ^2^ | **2.00E-02** |
| **Comorbidity and personality traits (n=349)** | **Cluster 1** | **Cluster 2** |  | **Statistical test** | ***p*** |
|  | (n=215; JM=.72) | (n=134; JM=.79) |  |  |  |
| *Mental disorders in DSM-IV (%)* |  |  |  |  |  |
| Borderline personality disorder | 5.1 | 24.6 | - | χ^2^ | **2.30E-07** |
| Psychotic disorders | 3.7 | 11.2 | - | χ^2^ | **1.20E-02** |
| Major depressive disorder | 29.8 | 41.8 | - | χ^2^ | **2.90E-02** |
| Antisocial personality disorder | 16.3 | 23.1 | - | χ^2^ | .15 |
| Anxiety disorder | 25.6 | 20.1 | - | χ^2^ | .3 |
| Attention deficit hyperactivity disorder | 22.3 | 21.6 | - | χ^2^ | .99 |
| *Zuckerman–Kuhlman Personality Questionnaire (ZKPQ); mean (SD)* |  |  |  |  |  |
| Neuroticism Anxiety personality factor | 10.18 (4.87) | 11.6 (5.02) | - | T-test | **9.40E-03** |
| Aggression Hostility personality factor | 8.74 (3.23) | 9.31 (3.17) | - | T-test | .11 |
| Sociability personality factor | 6.74 (3.3) | 6.17 (3.43) | - | T-test | .12 |
| Impulsive sensation seeking personality factor | 10.34 (4.3) | 10.79 (3.96) | - | T-test | .33 |
| Activity personality factor | 7.97 (3.59) | 7.85 (3.18) | - | T-test | .75 |
| Suicide attempt (%) | 16.7 | 97 | - | χ^2^ | **1.33E-47** |
| Suicide ideation (%) | 4.2 | 71.6 | - | χ^2^ | **4.93E-40** |
| Psychotic symptoms (%) | 55.8 | 67.9 | - | χ^2^ | **3.00E-02** |
| Sleeping disturbances (%) | 48.4 | 56.7 | - | χ^2^ | .16 |
| **Sociodemographic and health outcomes (n=307)** | **Cluster 1** | **Cluster 2** | **Cluster 3** | **Statistical test** | ***p*** |
|  | (n=60; JM=.60) | (n=140; JM=.60) | (n=107; JM=.64) |  |  |
| *EuropASI ^c^* |  |  |  |  |  |
| Legal status; median (IQR) | .00 (.00) | .00 (.00) | .20 (.50) | KWt | **1.60E-20** |
| Employment status; median (IQR) | .25 (.40) | .60 (.42) | .60 (.40) | KWt | **5.17E-11** |
| Medical status; median (IQR) | .00 (.03) | .10 (.70) | .00 (.50) | KWt | **5.90E-04** |
| Psychiatric status; mean (SD) | .31 (.19) | .40 (.23) | .40 (.22) | ANOVA | **2.00E-02** |
| Drug use; median (IQR) | .20 (.13) | .20 (.20) | .20 (.20) | KWt | .09 |
| Alcohol use; median (IQR) | .10 (.30) | .10 (.30) | .20 (.30) | KWt | .33 |
| Family/Social relationships; mean (SD) | .36 (.27) | .41 (.28) | .45 (.30) | ANOVA | .52 |
| *36-Item Short Form Survey (SF-36); mean (SD)* |  |  |  |  |  |
| Physical health | 52.33 (8.28) | 45.97 (1.98) | 45.93 (11.50) | Welch test | **1.00E-05** |
| Mental health | 37.76 (12.91) | 35.09 (13.99) | 35.25 (14.13) | ANOVA | .26 |
| Criminal record (%) | 6.7 | 0 | 100 | χ^2^ | **7.04E-64** |
| Unemployment (%) | 0 | 98 | 86 | χ^2^ | **3.00E-49** |
| Psychiatric hospitalizations ^b^ (%) | 8.3 | 22.1 | 15 | χ^2^ | **5.00E-02** |
| Psychiatric family history (%) | 53.3 | 45 | 36.4 | χ^2^ | .1 |
| Lifetime medical conditions (%) | 45 | 57.1 | 60.7 | χ^2^ | .13 |
| Substance use family history (%) | 65 | 57.1 | 60.7 | χ^2^ | .57 |
| Educational attainment (%) |  |  |  | ANOVA | .19 |
| 1 (Bachelor's degree or higher) | 13.3 | 10 | 8.4 |  |  |
| 2 (Secondary/High school) | 48.3 | 36.4 | 36.4 |  |  |
| 3 (Primary school) | 35 | 47.1 | 42.1 |  |  |
| 4 (Incomplete primary school) | 3.3 | 6.4 | 13.1 |  |  |

Note. For binary variables, we report the percentage of cases (yes) per cluster. For normally distributed continuous variables we report the mean and standard deviation (SD) and for non-normally distributed continuous variables we report the median and the interquartile range (IQR). In bold nominally significant associations.JM: Jaccard means; T-test: Pearson's T-test; MWU: Mann-Whitney U test; χ2: Chi-Squared test; KWt: Kruskal-Wallis H test.

a Logarithmic transformation was applied to continuous variables not following a normal distribution.

b For clearer interpretation of the data, we report the percentage of subjects having 1 or more counts per cluster.

c Scores ranging from 0 to 1 where rounded to 1 decimal and interpreted as ordinal variables with 11 levels.

**Supplementary Table 4**.

*Interaction between PGSs and lifetime emotional. physical and/or sexual abuse in the SUD phenome*

| **PGS** | **SUD-Phenome** | ***n ^a^*** | **Estimate** | **95% CI** | ***p*** |
| --- | --- | --- | --- | --- | --- |
| Attention-deficit hyperactivity disorder | Age at onset of substance use ^b^ | 701 | -.002 | -.04 , .04 | .94 |
|  | Years of substance use as proportion of lifespan | 631 | .32 | -2.57 , 3.2 | .83 |
|  | Antisocial personality disorder | 358 | 1.00 | .66 , 1.51 | .99 |
|  | Attention deficit hyperactivity disorder | 434 | .94 | .63 , 1.4 | .77 |
|  | Educational attainment | 701 | .96 | .73 , 1.26 | .77 |
|  |  |  |  |  |  |
| Anxiety | Number of outpatient treatments | 681 | 1.06 | .92 , 1.22 | .42 |
|  | Psychotic disorder | 180 | 1.10 | .61 , 1.99 | .76 |
|  | SF36 Physical health | 509 | .43 | -1.27 , 2.12 | .62 |
|  | Psychiatric family history | 524 | 1.03 | .73 , 1.46 | .86 |
|  |  |  |  |  |  |
| Bipolar disorder | Psychotic symptoms | 678 | 1.01 | .74 , 1.39 | .93 |
|  | Unemployment | 568 | 1.08 | .78 , 1.51 | .64 |
|  | Number of psychiatric hospitalizations | 504 | .87 | .53 , 1.42 | .57 |
|  | Substance use family history | 516 | .98 | .69 , 1.39 | .91 |
|  |  |  |  |  |  |
| Depression | Years between substance use and SUD | 691 | .96 | .74 , 1.25 | .78 |
|  | Number of outpatient treatments | 681 | 1.07 | .92 , 1.23 | .39 |
|  | ZKPQ-Neuroticism Anxiety personality factor | 492 | .20 | -.64 , 1.05 | .64 |
|  | ZKPQ-Aggression Hostility personality factor | 495 | -.28 | -.83 , .26 | .31 |
|  | Suicide attempt | 725 | .93 | .69 , 1.26 | .65 |
|  | Criminal record | 488 | .76 | .53 , 1.1 | .14 |
|  | Psychiatric family history | 524 | 1.31 | .92 , 1.86 | .13 |
|  |  |  |  |  |  |
| Post-traumatic stress disorder | Age at onset of substance use ^b^ | 701 | -.02 | -.06 , .03 | .45 |
|  | Number of inpatient detoxifications | 702 | .91 | .65 , 1.26 | .56 |
|  | Unemployment | 568 | .85 | .6 , 1.21 | .38 |
|  | Educational attainment | 701 | 1.08 | .81 , 1.44 | .58 |
|  |  |  |  |  |  |
| Schizophrenia | Age at onset of substance use ^b^ | 701 | .03 | -.01 , .08 | .11 |
|  | Psychotic disorder | 180 | .85 | .45 , 1.59 | .60 |
|  | Psychotic symptoms | 678 | .97 | .7 , 1.33 | .83 |
|  | Unemployment | 568 | .76 | .54 , 1.07 | .12 |
|  |  |  |  |  |  |
| Risk tolerance | Number of outpatient treatments | 681 | 1.12 | .96 , 1.31 | .14 |
|  | ZKPQ-Neuroticism Anxiety personality factor | 492 | .29 | -.6 , 1.18 | .53 |
|  | ZKPQ-Impulsive sensation seeking personality factor | 493 | .39 | -.37 , 1.14 | .32 |
|  | EuropASI-Legal status | 701 | 1.07 | .75 , 1.52 | .72 |
|  | Unemployment | 568 | .85 | .6 , 1.21 | .38 |
|  |  |  |  |  |  |
| Suicide attempt | Age at onset of SUD ^b^ | 699 | .00 | -.04 , .05 | .89 |
|  | Number of outpatient treatments | 681 | .98 | .84 , 1.13 | .77 |
|  | ZKPQ-Aggression Hostility personality factor | 495 | -.02 | -.58 , .55 | .96 |
|  | Psychotic symptoms | 678 | 1.03 | .75 , 1.41 | .86 |
|  | EuropASI-Legal status | 701 | 1.08 | .76 , 1.55 | .66 |
|  | EuropASI-Medical status | 699 | 1.13 | .84 , 1.51 | .41 |
|  | EuropASI-Psychiatric status | 701 | 1.35 | 1.03 , 1.78 | **2.94E-02** |
|  | Lifetime medical conditions | 705 | .95 | .7 , 1.31 | .76 |
|  | Educational attainment | 701 | 1.10 | .83 , 1.46 | .51 |
|  |  |  |  |  |  |
| Educational attainment | Age at onset of substance use ^b^ | 701 | .01 | -.03 , .06 | .50 |
|  | Age at onset of SUD ^b^ | 699 | .01 | -.03 , .06 | .54 |
|  | Number of therapeutic community interventions | 698 | .86 | .67 , 1.11 | .26 |
|  | Number of outpatient treatments | 681 | 1.06 | .91 , 1.23 | .47 |
|  | ZKPQ-Neuroticism Anxiety personality factor | 492 | .16 | -.73 , 1.06 | .72 |
|  | Criminal record | 488 | 1.27 | .86 , 1.89 | .23 |
|  | Unemployment | 568 | .73 | .51 , 1.04 | .08 |
|  | Substance use family history | 516 | 1.00 | .7 , 1.45 | .99 |
|  | Educational attainment | 701 | .94 | .71 , 1.26 | .69 |
|  |  |  |  |  |  |
| Well being | Number of outpatient treatments | 681 | 1.00 | .86 , 1.17 | .97 |
|  | EuropASI- Psychiatric status | 701 | 1.01 | .77 , 1.32 | .96 |
|  | EuropASI-Family/social relationships | 701 | .95 | .73 , 1.25 | .22 |
|  | Unemployment | 568 | .87 | .61 , 1.25 | .46 |
|  | Number of psychiatric hospitalizations | 504 | 1.23 | .73 , 2.07 | .44 |
|  | Substance use family history | 516 | 1.05 | .73 , 1.52 | .80 |

*Note.* Odds Ratio (OR) is reported for logistic regression and ordinal regression; Beta is reported for lineal regression; Incidence Rate Ratio (IRR) is reported for negative binomial regression.

^a^ For binary traits, with n1 individuals in category one and n2 individuals in the category two, sample size was calculated with the formula 4/(1/n1+1/n2).

^b^ Logarithmic transformations were applied to continuous variables not following a normal distribution.

**Supplementary Table 5**.

*Results from the association between the PGSs and the clinical clusters*

| **PGS** | **Clinical Clusters** | **OR** | **95% CI** | | ***p*** | |
| --- | --- | --- | --- | --- | --- | --- |
| Attention-deficit hyperactivity disorder | SUD related variables Cluster 2 | .94 | .79 , 1.13 | .54 | |  |
|  | Comorbidity and personality traits Cluster 2 | .87 | .7 , 1.07 | .19 | |  |
|  | Sociodemographic and health outcomes Cluster 2 | .97 | .71 , 1.31 | .82 | |  |
|  | Sociodemographic and health outcomes Cluster 3 | 1.19 | .87 , 1.62 | .28 | |  |
|  |  |  |  |  | |  |
| Anxiety | SUD related variables Cluster 2 | 1.11 | .92 , 1.33 | .28 | |  |
|  | Comorbidity and personality traits Cluster 2 | 1 | .81 , 1.23 | .98 | |  |
|  | Sociodemographic and health outcomes Cluster 2 | 1.19 | .87 , 1.63 | .28 | |  |
|  | Sociodemographic and health outcomes Cluster 3 | .97 | .71 , 1.34 | .87 | |  |
|  |  |  |  |  | |  |
| Bipolar disorder | SUD related variables Cluster 2 | .98 | .81 , 1.18 | .82 | |  |
|  | Comorbidity and personality traits Cluster 2 | 1.21 | .96 , 1.52 | .11 | |  |
|  | Sociodemographic and health outcomes Cluster 2 | 1.29 | .93 , 1.8 | .13 | |  |
|  | Sociodemographic and health outcomes Cluster 3 | 1.39 | .99 , 1.96 | .06 | |  |
|  |  |  |  |  | |  |
| Depression | SUD related variables Cluster 2 | .88 | .73 , 1.06 | .19 | |  |
|  | Comorbidity and personality traits Cluster 2 | 1.18 | .94 , 1.48 | .15 | |  |
|  | Sociodemographic and health outcomes Cluster 2 | 1.09 | .79 , 1.49 | .60 | |  |
|  | Sociodemographic and health outcomes Cluster 3 | 1.43 | 1.03 , 1.98 | **3.24E-02** | |  |
|  |  |  |  |  | |  |
| Post-traumatic stress disorder | SUD related variables Cluster 2 | 1.09 | .9 , 1.32 | .38 | |  |
|  | Comorbidity and personality traits Cluster 2 | 1 | .8 , 1.26 | .98 | |  |
|  | Sociodemographic and health outcomes Cluster 2 | 1.15 | .83 , 1.58 | .40 | |  |
|  | Sociodemographic and health outcomes Cluster 3 | 1.19 | .86 , 1.65 | .30 | |  |
|  |  |  |  |  | |  |
| Schizophrenia | SUD related variables Cluster 2 | .91 | .75 , 1.1 | .33 | |  |
|  | Comorbidity and personality traits Cluster 2 | 1 | .8 , 1.26 | .98 | |  |
|  | Sociodemographic and health outcomes Cluster 2 | 1.12 | .81 , 1.56 | .49 | |  |
|  | Sociodemographic and health outcomes Cluster 3 | 1.16 | .83 , 1.64 | .38 | |  |
|  |  |  |  |  | |  |
| Risk tolerance | SUD related variables Cluster 2 | 1.09 | .9 , 1.31 | .37 | |  |
|  | Comorbidity and personality traits Cluster 2 | 1.38 | 1.09 , 1.76 | **8.40E-03** | |  |
|  | Sociodemographic and health outcomes Cluster 2 | 1.29 | .93 , 1.8 | .13 | |  |
|  | Sociodemographic and health outcomes Cluster 3 | 1.53 | 1.09 , 2.15 | **1.35E-02** | |  |
|  |  |  |  |  | |  |
| Suicide attempt | SUD related variables Cluster 2 | 1.07 | .88 , 1.3 | .50 | |  |
|  | Comorbidity and personality traits Cluster 2 | 1.24 | .97 , 1.59 | .08 | |  |
|  | Sociodemographic and health outcomes Cluster 2 | 1.23 | .89 , 1.72 | .21 | |  |
|  | Sociodemographic and health outcomes Cluster 3 | 1.67 | 1.18 , 2.37 | **4.20E-03** | |  |
|  |  |  |  |  | |  |
| Educational attainment | SUD related variables Cluster 2 | .93 | .77 , 1.13 | .46 | |  |
|  | Comorbidity and personality traits Cluster 2 | .98 | .77 , 1.24 | .84 | |  |
|  | Sociodemographic and health outcomes Cluster 2 | .85 | .62 , 1.19 | .35 | |  |
|  | Sociodemographic and health outcomes Cluster 3 | .57 | .4 , .81 | **1.80E-03** | |  |
|  |  |  |  |  | |  |
| Well being | SUD related variables Cluster 2 | 1.18 | .96 , 1.44 | .11 | |  |
|  | Comorbidity and personality traits Cluster 2 | 1.17 | .92 , 1.48 | .20 | |  |
|  | Sociodemographic and health outcomes Cluster 2 | .8 | .58 , 1.12 | .20 | |  |
|  | Sociodemographic and health outcomes Cluster 3 | .74 | .52 , 1.04 | .09 | |  |

*Note*. Multinomial regression performed using cluster 1 as the reference. In bold nominally significant associations. OR = Odds Ratio.

***References***

1. Demontis D, Walters RK, Martin J, Mattheisen M, Als TD, Agerbo E, et al. Discovery of the first genome-wide significant risk loci for attention deficit/hyperactivity disorder. Nat Genet. 2019;51:63–75.

2. Mullins N, Forstner AJ, O’Connell KS, Coombes B, Coleman JRI, Qiao Z, et al. Genome-wide association study of more than 40,000 bipolar disorder cases provides new insights into the underlying biology. Nat Genet. 2021;53:817–829.

3. Howard DM, Adams MJ, Clarke TK, Hafferty JD, Gibson J, Shirali M, et al. Genome-wide meta-analysis of depression identifies 102 independent variants and highlights the importance of the prefrontal brain regions. Nat Neurosci. 2019;22:343–352.

4. Nievergelt CM, Maihofer AX, Klengel T, Atkinson EG, Chen CY, Choi KW, et al. International meta-analysis of PTSD genome-wide association studies identifies sex- and ancestry-specific genetic risk loci. Nat Commun. 2019;10.

5. Trubetskoy V, Pardiñas AF, Qi T, Panagiotaropoulou G, Awasthi S, Bigdeli TB, et al. Mapping genomic loci implicates genes and synaptic biology in schizophrenia. Nature. 2022;604:502.

6. Karlsson Linnér R, Biroli P, Kong E, Meddens SFW, Wedow R, Fontana MA, et al. Genome-wide association analyses of risk tolerance and risky behaviors in over 1 million individuals identify hundreds of loci and shared genetic influences. Nat Genet. 2019;51:245–257.

7. Docherty AR, Mullins N, Ashley-Koch AE, Qin XJ, Coleman J, Shabalin AA, et al. Genome-wide association study meta-analysis of suicide attempt in 43,871 cases identifies twelve genome-wide significant loci. MedRxiv. 2022. 2022. https://doi.org/10.1101/2022.07.03.22277199.

8. Lee JJ, Wedow R, Okbay A, Kong E, Maghzian O, Zacher M, et al. Gene discovery and polygenic prediction from a genome-wide association study of educational attainment in 1.1 million individuals. Nat Genet. 2018;50:1112–1121.

9. Baselmans BML, Jansen R, Ip HF, van Dongen J, Abdellaoui A, van de Weijer MP, et al. Multivariate genome-wide analyses of the well-being spectrum. Nat Genet. 2019;51:445–451.
